# The Utility of Cyclodextrin for Countering μ-Opioid Receptor Drug Overdoses

**DOI:** 10.1021/acscentsci.4c01990

**Published:** 2024-12-10

**Authors:** Noriko Ogawa

**Affiliations:** Department of Pharmacy, College of Pharmacy, Kinjo Gakuin University, 2-1723 Omori, Moriyama-ku, Nagoya, Aichi 463-8521, Japan

**Figure 1:**
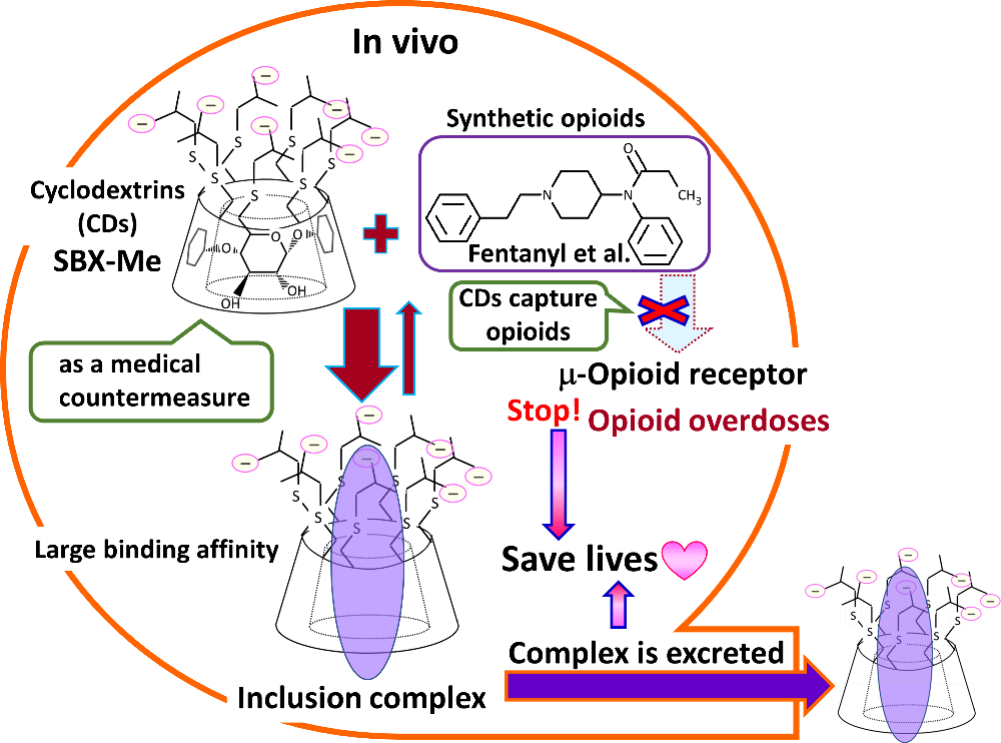
Cyclodextrins save lives by trapping synthetic opioids in vivo.

Cyclodextrins (CDs) are cyclic, nonreducing oligosaccharides linked through α-1,4 glycosidic bonds, with a hydrophilic exterior and a hydrophobic, cavity-like interior. CDs can incorporate a hydrophobic guest compound into the cavity to form inclusion complexes, and since the 1970s have been widely used as pharmaceutical excipients for the solubilization and stabilization of active pharmaceutical ingredients (APIs).^1^ There has been increasing effort recently to use CDs as APIs exhibiting novel pharmacological properties. In this issue of *ACS Central Science*, Malfatti, Valdez, and co-workers report a new cyclodextrin derivative, Subetadex-α-methyl (SBX-Me), as a medical countermeasure for fentanyl and related opioid overdoses (Figure 1).^2^

Fentanyl is an important therapeutic drug used for anesthesia and analgesia, but it is also increasingly used illegally, which has led to a rise in deaths from opioid overdoses.^3^ Thus, there is a need to develop medical countermeasures for exposure to fentanyl and related opioids. In SBX-Me, the primary hydroxyl group of β-CD is replaced with a 2-methyl-3-thiopropanoic acid group. SBX-Me shortened the recovery time of rats exposed to opioid drugs, from approximately 35 to 17 min for fentanyl, from approximately 172 to 59 min for carfentanil, and from approximately 18 to 12 min for remifentanil.^2^ The findings reported herein suggest that SBX-Me represents a new type of therapeutic agent and mechanism of action and could mitigate the number of overdose deaths.

CDs have long been used as pharmaceutical additives. Recent attempts to expand the utility of CDs have mainly focused on the structural and functional properties of CDs to either 1) scavenge toxic compounds or 2) help regulate cholesterol levels. The formation of CD-target compound inclusion complexes can prevent accumulation of the target compound, counteract direct interactions of the compound with tissues, alter the pharmacokinetics of the compound, and facilitate excretion of the compound from the body.^4^ The use of CDs for detoxification was first described in 1983^5^ and was utilized in clinical life-saving activities in 1987.^6^ The CD derivative Sugammadex was approved in 2008 as the first CD-based API.^4,7^ Sugammadex is a γ-CD derivative that forms an inclusion complex with the muscle relaxant rocuronium bromide to reduce the concentration of this muscle relaxant at the neuromuscular junction, thereby aiding rapid recovery from muscle relaxation.^7^ Sugammadex was the first and still the only CD approved as an API and is a blockbuster drug. The possibility that SBX-Me might be the next CD-based blockbuster drug is driving intense research into the utility of SBX-Me for capturing fentanyl before it binds to μ-opioid receptors in the central nervous system. This paper reports that the administration of SBX-Me shortens the recovery time from fentanyl and related drugs in rats. SBX-Me might affect the toxicity profile of these synthetic opioids by sequestering free opioids and preventing the drug from interacting with the target receptors, thereby neutralizing the effect of the opioid. This proposed mechanism differs from that of conventional treatments, in which competitive antagonists such as naloxone compete with opioids for receptor binding sites but is similar to the pharmacological mechanism of action of Sugammadex.^2^

The strength of the interaction between SBX-Me and synthetic opioids such as fentanyl will largely determine whether SBX-Me can be used as a drug. The stability constant reflects the strength of the interaction between a CD and a guest compound. The stability constant for Sugammadex complexed with rocuronium is approximately 10^7^ M^–1^, as determined by isothermal titration calorimetry, and approximately 750,000 M^–1^ as determined by NMR titration experiments.^8,9^ These large binding affinities are unusual for small molecule host–guest interactions. An extremely high stability constant is detrimental to the use of CDs as pharmaceutical additives for solubilization, etc. because the stable complex can block the effect of the drug. However, such highly stable complexes are attractive when CDs are used as scavengers. Valdez and co-workers previously reported Subetadex+1 (SBX+1), a β-CD derivative whose structure is similar to that of SBX-Me but whose primary hydroxyl group is replaced with 4-mercaptobutanoyl. SBX+1 has a maximum binding affinity with fentanyl of *K*_a_ = 66,500 M^–1^ (NMR titration experiment), which is the highest stability constant of the compounds previously reported by the authors.^9^ The stability constant of SBX-Me complexed with fentanyl was not provided in the study presented herein, but even if it is similar to that of SBX+1, it would be less than one-tenth of the stability constant of Sugammadex complexed with rocuronium. It is therefore unknown if the stability constant of SBX-Me complexed with fentanyl is sufficiently high for it to be useful as a scavenger. Fentanyl and carfentanil were detected in brain tissue when administered together with SBX-Me to rats,^2^ whereas SBX-Me was not. SBX-Me might be unable to completely capture fentanyl and related compounds before they cross the blood-brain barrier. The administration of SBX-Me extends the elimination half-life of fentanyl from 5.37 to 6.42 h and that of remifentanil from 8.24 to 9.74 h, thus extending the residence time of these drugs in rats.^2^ It is therefore important to determine whether the intact SBX-Me/opioid complex is excreted. Further research, including structural improvements, could transform SBX-Me into a more effective and safer drug. SBX-Me has a chiral center in the thioalkyl carboxylate chain of its substituent. The SBX-Me used in the present study is a mixture of several isomers.^2^ More accurate stability constants could be measured by using enantiomerically pure compounds, and useful information might be obtained by comparing optical isomers.

The use of SBX-Me to capture fentanyl and decrease fatal overdoses is very challenging because the interaction between fentanyl and the μ-opioid receptor remains poorly understood.^10^ SBX-Me holds promise as a powerful opioid binder that could save many lives.

Necessary future research includes elucidating and improving the stability constants of SBX-Me with fentanyl and related compounds, understanding the mechanism of interaction, determining the single crystal structures of the inclusion complex formed by SBX-Me and fentanyl, and conducting safety studies.
